# Identification of the robust predictor for sepsis based on clustering analysis

**DOI:** 10.1038/s41598-022-06310-8

**Published:** 2022-02-11

**Authors:** Jae Yeon Jang, Gilsung Yoo, Taesic Lee, Young Uh, Juwon Kim

**Affiliations:** 1 Division of Hematology-Oncology, Department of Internal Medicine, Yonsei University Wonju College of Medicine, Wonju, South Korea; 2 Department of Laboratory Medicine, Yonsei University Wonju College of Medicine, 162 Ilsan-dong, Wonju-city, 220-701 Gangwon-do South Korea; 3 Department of Family Medicine, Yonsei University Wonju College of Medicine, Wonju, South Korea; 4 Center for Precision Medicine and Genomics, Wonju Severance Christian Hospital, Wonju, South Korea

**Keywords:** Biomarkers, Infectious diseases, Risk factors

## Abstract

Sepsis is a life-threatening disorder with high incidence and mortality rate. However, the early detection of sepsis is challenging due to lack of specific marker and various etiology. This study aimed to identify robust risk factors for sepsis via cluster analysis. The integrative task of the automatic platform (i.e., electronic medical record) and the expert domain was performed to compile clinical and medical information for 2,490 sepsis patients and 16,916 health check-up participants. The subjects were categorized into 3 and 4 groups based on seven clinical and laboratory markers (Age, WBC, NLR, Hb, PLT, DNI, and MPXI) by K-means clustering. Logistic regression model was performed for all subjects including healthy control and sepsis patients, and cluster-specific cases, separately, to identify sepsis-related features. White blood cell (WBC), well-known parameter for sepsis, exhibited the insignificant association with the sepsis status in old age clusters (K3C3 and K4C3). Besides, NLR and DNI were the robust predictors in all subjects as well as three or four cluster-specific subjects including K3C3 or K4C3. We implemented the cluster-analysis for real-world hospital data to identify the robust predictors for sepsis, which could contribute to screen likely overlooked and potential sepsis patients (e.g., sepsis patients without WBC count elevation).

## Introduction

Sepsis is a life-threatening disorder caused by overwhelming immune response to infection^1^. In 2017, the incidence of sepsis worldwide were estimated 48.9 million cases (95% uncertainty interval [UI] 38.9–62.9), and 11.0 million (95% UI: 11.8–54.5) cases of sepsis-related death has been reported, accounting for about 20% of all global deaths^2^. In Korea, the incidence of sepsis was 265.7 (95% confidence interval [CI], 254.7–277.1) per 100,000 person-years in 2005 and 453.1 (95% CI, 439.0–467.5) in 2012^3^. Moreover, the 6-month mortality rate among sepsis patients in Korea was 26.5% (95% CI, 24.4% to 28.8%) in 2005 and 30.1% (95% CI, 28.4% to 31.9%) in 2012^3^.

The diagnostic criteria of Systemic inflammatory response syndrome (SIRS), which is used for identifying sepsis^4^, includes four parameters: presence of fever, tachycardia, tachypnea, and leukocytosis or leukopenia. Albeit the ease of collection of information on those four parameters in clinical setting, these diagnostic markers are non-specific. Furthermore, laboratory markers used for the diagnosis of sepsis have low screening power in specific population group. For example, Seigel et al.^5^ pinpointed that about 52% of patients with bacteremia had a normal range of WBC levels, and 21% of patients diagnosed with severe sepsis or septic shock manifested a normal WBC count at the time of admission to the emergency department. As a consequence, there have been tremendous efforts to find the sensitive and suitable marker that would be used for early diagnosis of sepsis, including C-reactive protein (CRP)^6–9^, procalcitonin (PCT)^6,7,10–14^ and interleukins^8,13,15,16^. In the early years, erythrocyte sedimentation rate (ESR) and CRP as markers of inflammation were used in diagnosis of sepsis, however, these markers have limitations due to lack of specificity for sepsis. PCT, a 116-amino-acid peptide, has shown to be strongly associated with systemic bacterial infection and the prognosis of the disease, but the cost for testing is relatively high and not frequently used for screening the disease.

Numerous factors, such as age, gender, and presence of chronic diseases, have been reported to influence sepsis-like immune responses^17–24^ and the infection origins of different organs have resulted in rather heterogeneous clinical outcomes. Previously, Knox et al.^25^ attempted to identify several sepsis groups susceptible to multiple organ dysfunction syndrome using cluster analysis. Considering that determination of robust or group-specific biomarkers may be a crucial strategy for early detection of sepsis, the identification of potential predictors for sepsis in heterogeneous subgroups would be meaningful. Therefore, in this study, we conducted the cluster analysis and identified robust or cluster-specific risk factors for sepsis using hematology indices.

## Materials and methods

### Study population

We conducted a cross-sectional study analyzing about 20,000 healthy subjects or patients who visited the Wonju Severance Christian Hospital (WSCH) from January 2017 to December 2020. In detail, subjects were collected using two different sources including Laboratory Medical System and health check-up database from Electronic Medical records (EMR) at WSCH. For screening sepsis patients, three doctors reviewed the ICD-10 code matched to sepsis or SIRS (Table S1). Afterwards, database administrator constructed the dataset including patients diagnosed to sepsis based on the ICD codes from the EMR at WSCH. For healthy control samples, individuals who had undergone health check-up were also collected by the same database administrator. The patients with negative results of blood culture tests and SIRS (ICD 10) were excluded. Then, following subjects were eliminated: those who aged under 19 years or having missing values for laboratory results; who were not diagnosed with sepsis diagnosis at the time of admission; who were diagnosed to rheumatologic diseases, malignancy, and hematologic diseases; and who received immunosuppressant. Finally, a total of 2,490 sepsis patients and 16,916 health check-up participants were enrolled for the analyses of this study. This study was conducted in accordance with the principles of the Declaration of Helsinki and approved by the Institutional Review Board (IRB) of WSCH (No. CR321107). Because this study was performed retrospectively based on existing medical records, the requirement for written consent from the patients was waived, which was approved by IRB (No. CR321107).

### Definition of sepsis

We defined sepsis patients as follows: the patients with (1) the ICD-10 code for sepsis; and (2) the ICD-10 code for SIRS and exhibited bacteremia (manually checked by domain expert). Bacteremia was defined using the criteria of the US centers for Disease Control and Prevention/National Healthcare Safety Network surveillance^26^. The criteria of bacteremia are as follows: (1) the pathogen was identified in one or more blood culture tests; (2) the pathogen was not considered common contaminants.

### Measurements

The hematology indices, Delta neutrophil index (DNI) and Myeloperoxidase index (MPXI) were estimated in an automated hematology analyzer ADVIA2120 (Siemens, Tarrytown, NY) following the manufacturer’s recommendation. The DNI was calculated using the following formula: DN (%) = (the leukocyte subfraction assayed in the MPO channel by cytochemical reaction) − (the leukocyte subfraction counted in the nuclear lobularity channel by reflected light beam)^27^. The MPXI was determined with the blood auto-analyzer using 4-chloro-1-naphthol (an MPO substrate in granulocytes), and in these cells black precipitates were formed. As stained white blood cells (WBC) pass through the flow cell, light scatter (y-axis) and absorbance (x-axis) are measured by a tungsten-halogen light source, and the MPXI is defined by the deviation from the mean neutrophil values on the x-axis^28^.

### Cluster analysis

We conducted cluster analysis to identify cluster-specific factors related to sepsis status. Before performing the cluster analysis, we scaled neutrophil–lymphocyte ratio (NLR) and DNI by log-transformation to reduce the effect of outliers (Figure S1). Afterwards, we scaled all continuous variables (*V*) by using z-normalization as follow.

$$\text{Scaled variable }\left({\text{V}}\right) = \frac{{\text{V}}\text{ (an individual subject)}-\text{average of }{\text{V}}\text{ (all subjects)}}{\text{standard deviation of }\text{V }\text{(all subjects)}}$$

In the medical field, numerous clustering methods have been implemented to reveal biological and pathophysiological findings. Among them, K-means clustering is widely used in various fields, including clinical medicine and bioinformatics^29–31^. Ahlqvist et al.^29^ applied K-means clustering algorithm to patients with newly diagnosed diabetes, identifying five novel clusters that exhibit different associational patterns with the risk of diabetic complication. Kwon et al.^31^ also used the K-means clustering method to yield five sub-groups and their cluster-specific risk factors for sarcopenia. Motivated by these studies, we performed the K-means clustering algorithm to identify the robust or hidden (but potential) risk factors for sepsis.

The determination of features for the cluster analysis is a crucial task. Typically, domain experts or groups have empirically determined the risk factors to be related with the interest disease as the criteria for the cluster analysis^29,31^. For example, Ahlqvist et al.^29^ established five clusters based on six clinical variables that are known to be related with diabetes. Similarly, Kwon et al.^31^ made five sub-groups according to nine lifestyle-related features known to be associated with the low muscle mass. Taken together, hematologist and laboratorian had empirically determined seven variables for the cluster analysis based on the literature-based review (Table S2).

Another main task of the K-means clustering is to select *k*, which means the number of groups to be categorized. Ahlqvist et al.^29^ determined the optimal *k* based on silhouette width, besides Kwon et al.^31^ empirically determined *5* as the *k*. Based on these studies, we empirically determined the number of clusters, and performed the cluster analysis twice according to the change of *k* from three to four.

We used both healthy participants and sepsis patients for the establishment of clusters. However, due to different characteristics between healthy subjects and sepsis patients, the biased arrangement of them into each cluster was shown. For example, in an experiment (data not shown), a cluster consisted of about 2,000 subjects, of which 1,922 were septic patients. To overcome this problem, we used ComBat algorithm^32,33^ to reduce differential signatures between healthy subjects and septic patients, thereby, yielding more generalized subjects. Afterward, we established three to four clusters that had similar characteristics among subjects in the same cluster for the eight variables through the K-means algorithm with setting 100,000 as iteration.

### Statistical analysis

Student’s t-test and Chi square test were used to analyze differential characteristics of continuous and categorical variables according to two conditions (i.e., normal and sepsis subjects), respectively. We used a one sample *t*-test to characterize each cluster based on seven variables. We used a logistic regression model to uncover whether a predictor (i.e., independent variable) is related to the sepsis status (i.e., dependent variable: presence vs. absence). In the multivariate model, seven variables (i.e., age, WBC count, NLR, hemoglobin, platelet count, DNI, and MPXI) were used as both independent and confounding variables, and a variable (i.e., sex) was solely used as confounder. We conducted all statistical analyses and illustrations using the R language (version 4.0.1).

## Results

### Clinical and laboratory characteristics of the study participants

The general characteristics of the enrolled subjects in this study are summarized based on the status of sepsis in Table 1. Subjects with the diagnosis of sepsis were significantly older than those without sepsis. The WBC, neutrophil count, NLR, DNI, and MPXI were higher in patients diagnosed with sepsis while other laboratory indices were all lower in the sepsis group.

**Table 1:** General characteristics of study population based on sepsis status.

	Normal group	Sepsis group	P value
n	16,916	2,490	
Age, year	49 ± 0.08	73.3 ± 0.27	< 0.001
Male, n	10,666 (63.1)	1277 (51.3)	< 0.001
WBC count, 10^9^/L	5.5 ± 0.01	13.5 ± 0.18	< 0.001
Neutrophil count, 10^9^/L	3.1 ± 0.01	11.8 ± 0.17	< 0.001
Lymphocyte, 10^9^/L	1.799 ± 0.01	1.001 ± 0.02	< 0.001
NLR	1.8 ± 0.01	19.3 ± 0.43	< 0.001
Hemoglobin, g/dL	14.7 ± 0.01	11.4 ± 0.05	< 0.001
Platelet, 10^9^/L	250 ± 0.44	210.4 ± 2.62	< 0.001
DNI	0.1 ± 0.01	9.1 ± 0.25	< 0.001
MPXI	−0.1 ± 0.03	2.7 ± 0.12	< 0.001

Continuous and categorical variables are described as mean ± standard error and frequency (percent), respectively. Abbreviations: WBC, white blood cell; NLR, neutrophil–lymphocyte ratio; DNI, delta neutrophil index; MPXI, Myeloperoxidase Index; n, number.

### Characteristics of each of the three or four clusters

**Figure 1:**
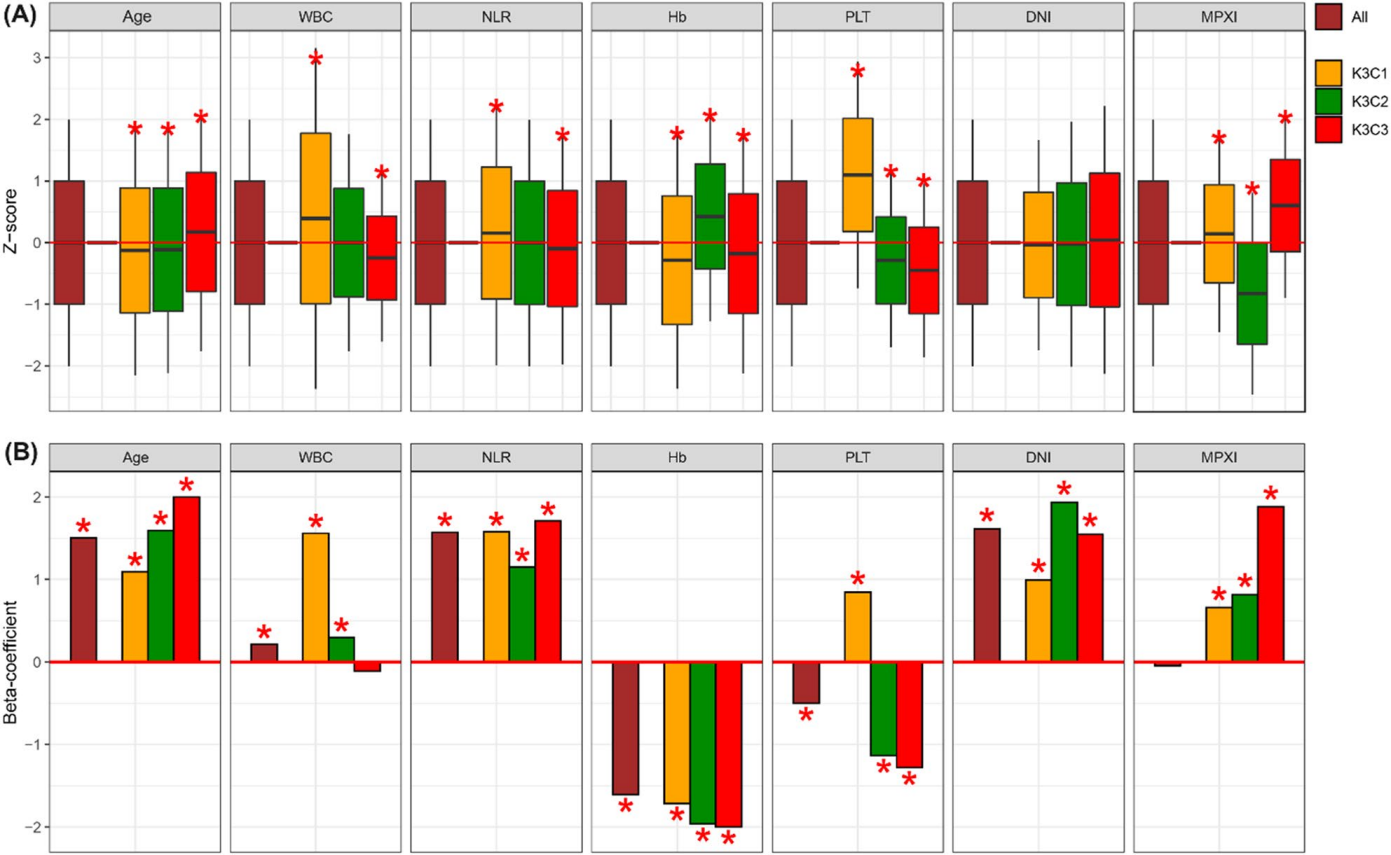
Three subgroups established by K-means clustering. (**A**) Three subgroups were characterized based on seven sepsis-related variables using one sample *t*-test. The brown box plot is about all subjects including healthy control and sepsis patients. Black middle, upper margin, and max lines in each boxplot indicate means, one standard deviation (SD), and 2-SD levels. (**B**) Beta-coefficients were measured from multivariate logistic regression. Brown bar was made by logistic regression for all subjects. Other bars (yellow, green, red) were curated by logistic regression for each cluster-specific subjects. A red asterisk indicates *p*-value less than 0.001.

We constructed three or four clusters (C) each for both healthy subjects and sepsis patients using the K-means clustering. Each group (i.e., cluster) included participants with similar characteristics for seven variables. Also, we performed two clustering tasks with grouping all subjects into three or four groups. In case of K-means clustering with setting three as K, 4,941, 6,608, and 7,877 subjects were grouped to three clusters (K3C1, K3C2, and K3C3), respectively. The biochemical characteristics of the three clusters were characterized based on one sample *t*-test (Fig. 1A). For example, the participants in K3C3 were characterized as relatively old age, low levels of WBC counts, NLR, hemoglobin concentration, platelet counts, and high DNI levels (Fig. 1A).

**Figure 2:**
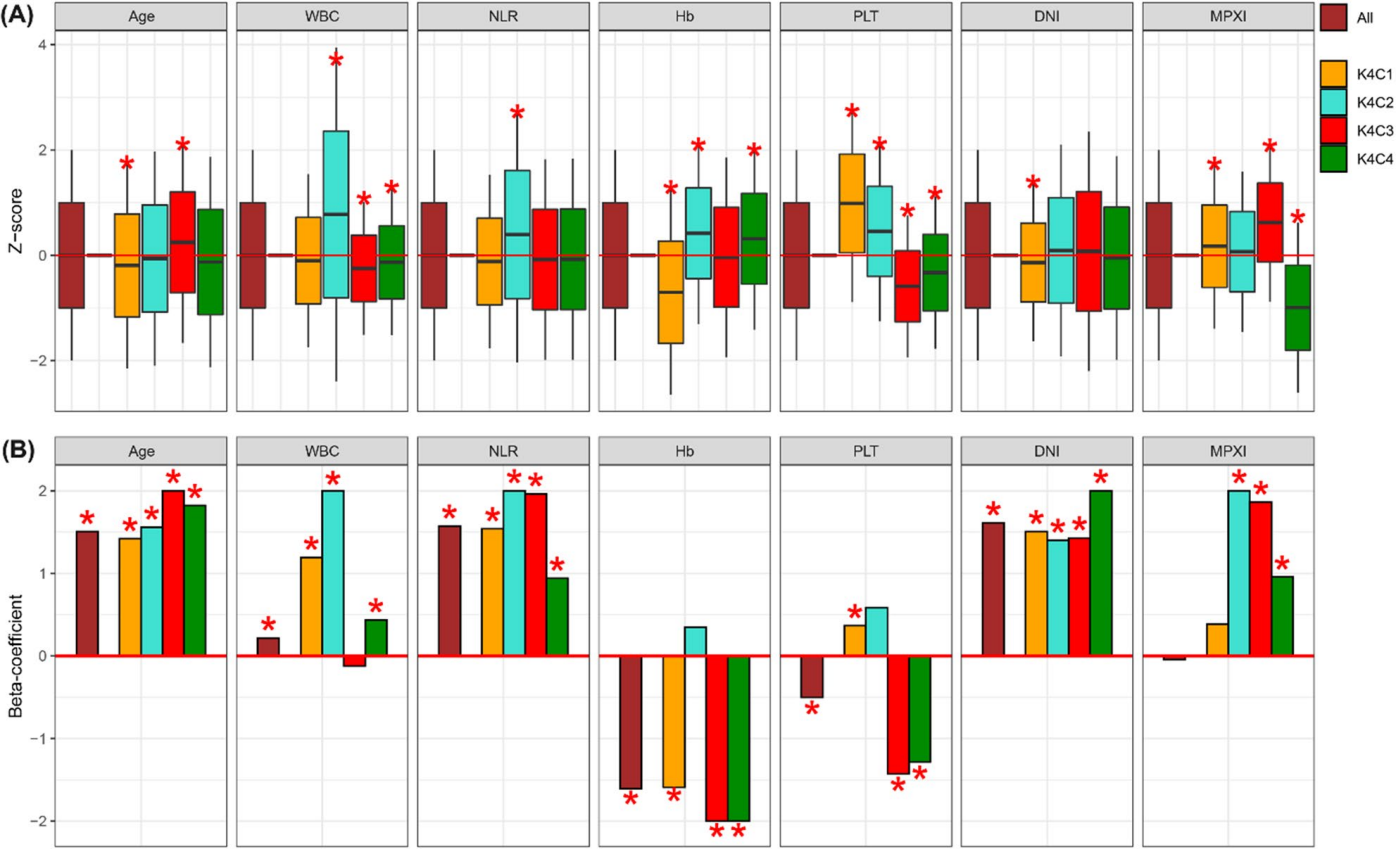
Four subgroups established by K-means clustering. (**A**) Four subgroups were characterized based on seven sepsis-related variables using one sample *t*-test. The brown box plot is about all subjects including healthy control and sepsis patients. Black middle, upper margin, and max lines in each boxplot indicate means, one standard deviation (SD), and 2 SD deviation levels. (**B**) Beta-coefficients were measured from multivariate logistic regression. Brown bar was made by logistic regression for all subjects. Other bars (yellow, light cyan, red, and green) were curated by logistic regression for each cluster-specific subjects. A red asterisk indicates p-value less than 0.001.

In case of the clustering model with setting four as K, 4,061, 3,541, 6,679, and 5,145 were arranged to K4C1, K4C2, K4C3, and K4C4, respectively. Among them, the notable cluster was the K4C3 which included subjects that are relatively old and have low levels of WBC and platelet counts, and high level of MPXI (Fig. 2A). When setting p-value of 0.001 as the cut-off, the statistical significance for the biasness measured via one sample *t*-test of following variables disappeared in K4C3 compared to K3C3: NLR and hemoglobin.

### Comparison of general and cluster-specific risk factors

We performed multivariate logistic regression to identify risk factors significantly related to the presence of sepsis for both all and cluster-specific subjects. For three clusters made by the K(= 3)-means clustering, age, NLR, hemoglobin, and DNI levels were significantly associated with the sepsis status in both entire and three clusters-specific subjects (Fig. 1B). The subjects in K3C3 had the insignificant association between WBC count and the presence of sepsis, besides the significant relationship between DNI level and the sepsis status. Other laboratory markers significantly related to the sepsis status in K3C3 were NLR, hemoglobin, platelet, DNI, and MPXI (Fig. 1B).

In case of four groups constructed by the K(= 4)-means clustering, age, NLR, and DNI levels were the robust factors significantly related with the presence of sepsis in both all and cluster-specific subjects. In K4C3 cluster, WBC count was not a risk factor for the sepsis status whilst NLR, hemoglobin, platelet, DNI, and MPXI levels were significant predictors (Fig. 2B). Taken together, NLR and DNI were the robust predictors in all subjects as well as three or four cluster-specific subjects.

**Figure 3:**
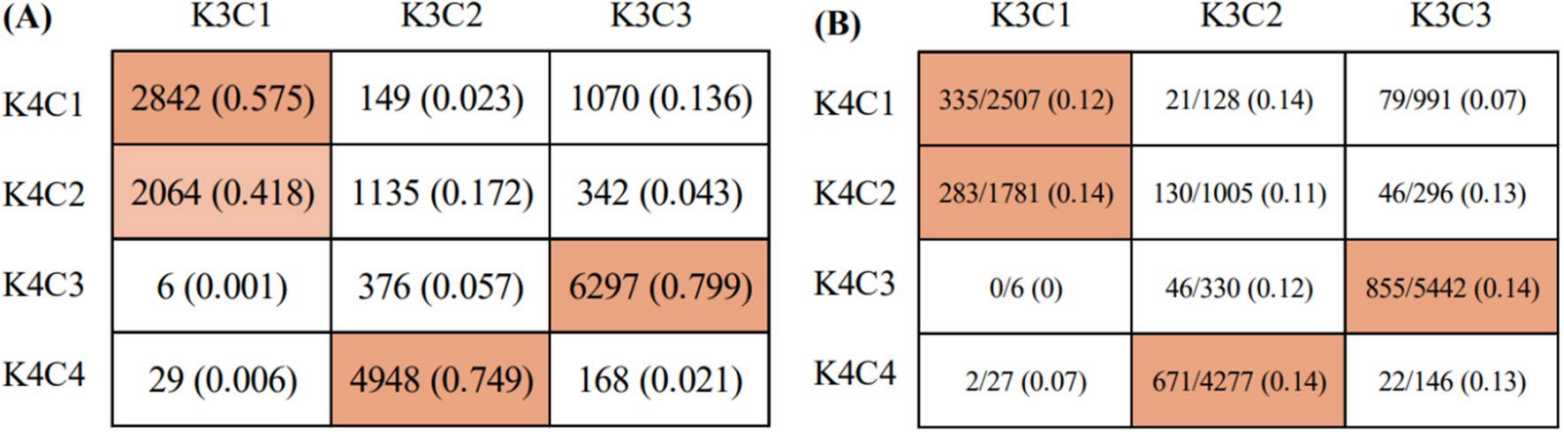
Comparison between three and four clusters established by K-means clustering. Colored rectangles indicate cases with prominently high number of common subjects between two subgroups. (**A**) Results are described number and column-based ratio for subjects including healthy control and sepsis patients in common between two subgroups. (**B**) Results are described as number of sepsis patients/healthy cases and ratio [sepsis patients/(sepsis + healthy subjects)].

We compared the distribution of subgroup-specific subjects arranged by K(= 3)-means clustering with that by K(= 4)-means clustering. 79.9% (6,297/7,877) of K3C3 subjects were included in K4C3, and 85.3% (855/1,002) of sepsis patients in K3C3 were arranged into K4C3 (Fig. 3).

## Discussion

We conducted clustering analysis to categorize all subjects (healthy controls and sepsis patients) into three to four groups based on seven clinical and laboratory markers. Then, the associational analysis using multivariate regression model was performed for all subjects and cluster-specific cases. As a result, age, NLR, and DNI were the robust predictors for the sepsis status in all subjects as well as cluster-specific groups.

The WBC levels in elderly subjects (K3C3 and K4C3) exhibited insignificant association with the sepsis status. Aminzadeh et al.^34^ reported only 60% and 6% sepsis subjects had WBC ≥ 14,000 and < 4,000 WBC counts, respectively. In a study by Caterino et al.^35^, the predictive performance of WBC was poor for the classification of sepsis (area under the curve: 0.5; 95% CI: 0.3 to 0.7). In elderly patients, as the aging progresses, chronic inflammatory status is pervasive, and cell regeneration ability and phagocytosis are reduced. For these reasons, the response of neutrophils to infection can be delayed^36–38^. Therefore, normal neutrophil count should not be allowed to exclude patients from being diagnosed with sepsis in elderly population.

Several studies have reported the DNI level as predictors^39–44^ for sepsis and sepsis-related mortality. Seok et al.^42^ reported 73.4% [95% confidence interval (CI): 64.9–80.9] and 97.9% [95% CI: 88.7–99.9] of sensitivity and specificity, respectively, when setting 2.7 of DNI as cut-off, indicating that DNI is a biomarker with high negative predictive value. Park et al.^41^ specified subjects diagnosed with severe sepsis or septic shock, and resulted 88.6% and 84.7% of negative predictive value (NPV) and positive predictive value (PPV), respectively, when setting 6.5 of DNI as the cut-off. In addition, several studies have suggested the DNI as prognostic factor for sepsis-related mortality^39^.

NLR has been considered as an easily obtainable parameter to assess the inflammatory status of patients. It is known to be a useful predictor for the adverse events (e.g., mortality) of patients experiencing diabetes^45^, cancer^46–48^, and abdomen operation^49,50^. Specifically, NLR exhibited the significant association with sepsis in both all subjects and cluster groups. NLR have been known to be biomarker for the diagnosis of sepsis^51^. Moreover, several expert groups have suggested the NLR as a potential prognostic factor for the sepsis-related complication or mortality^51–53^.

Clustering is an establishment task to arrange heterogeneous samples or variables to several groups, in which each group includes homogenous subjects or variables. Many methods, including K-means clustering, auto-encoder^30^, and NMF, have been used to construct groups, clusters, or modules. Moon and Lee developed the integrative non-linear representation method (Joint deep semi-NMF, JDSNMF) based on deep learning and modified NMF considering both intensity and direction of feature for the clustering task^54^. We implemented a widely used and simple method, K-means clustering to establish several subgroups. Future study implementing integrative algorithm^30,54^ for clustering is needed to establish the sophisticated module.

Our study is a multidiscipline work including the database professional, laboratorian, computer analyst, and domain expert (i.e., Hemato-oncologist). Data analyzed in this study is automatically curated from real-world hospital database by database professional. Afterward, laboratorian and domain expert empirically determined candidate variables that have potential relationship with the sepsis status. Crucial limitation of the present study is the determination of sepsis status based on ICD codes and blood culture results retrospectively. Therefore, it is crucial to establish the prediction model using robust predictors for sepsis status and apply this in real-world setting^55,56^.

In conclusion, this study revealed that the DNI and NLR are promising diagnostic marker for sepsis in specific group that exhibited insignificant association between WBC count and sepsis status. These findings could contribute to screen sepsis patients without leukocytosis in the emergency care units.

## Supplementary Information

Supplementary Information.
