# Supplementary material for: Identification of the robust predictor for sepsis based on clustering analysis
Source: Sci Rep. 2022 Feb 11;12:2336. doi: 10.1038/s41598-022-06310-8 (PMC8837750; doi:10.1038/s41598-022-06310-8)
Supplement: Supplementary file 1 — Supplementary Information. [file 41598_2022_6310_MOESM1_ESM.pdf]

Identification of the robust predictor for sepsis based on clustering analysis

(Supplementary Materials)

Jae Yeon Jang[1,†], Gilsung Yoo[2,†], Tae Sic Lee[3], Young Uh[2], Juwon Kim[2,4,*]

[1] Division of Hematology-Oncology, Department of Medicine, Wonju Christian Hospital, Yonsei University College of Medicine, Wonju, Korea.

[2] Department of Laboratory Medicine, Yonsei University Wonju College of Medicine, Wonju, South Korea.

[3] Department of Family Medicine, Yonsei University Wonju College of Medicine, Wonju, South Korea.

[4] Center for Precision Medicine and Genomics, Wonju Severance Christian Hospital, Wonju, South Korea

Supplementary Table 1. ICD codes matched to Sepsis

Disease	ICD 10 code
Sepsis	A4*, R65102, R572
SIRS	R65*

Supplementary Table 2. Literature-based search for identification of sepsis-related variables

	Evidence
Age	Butcher et al., 2000<br>Martin-Loeches et al., 2019<br>Weiskopf et al., 2009<br>Wenisch et al., 2000)
WBC	Bone et al., 1992<br>Farkas, 2020
Hb	Bateman et al., 2017<br>Farkas, 2020<br>Jiang et al., 2019<br>Piagnerelli et al., 2007
Platelet	Assinger et al., 2019<br>Guclu et al., 2013<br>Vardon-Bounes et al., 2019
NLR	de Jager et al., 2010<br>Huang et al., 2020<br>Liu et al., 2016
DNI	Ahn et al., 2018<br>Celik et al., 2019<br>Kim et al., 2017<br>Park et al., 2011<br>Park et al., 2020<br>Seok et al., 2012
MPXI	Cha et al., 2016<br>Cha et al., 2015<br>Yonezawa et al., 2010

Supplementary Figure 1. The change of distribution of NLR and DNI before and after log-transformation.

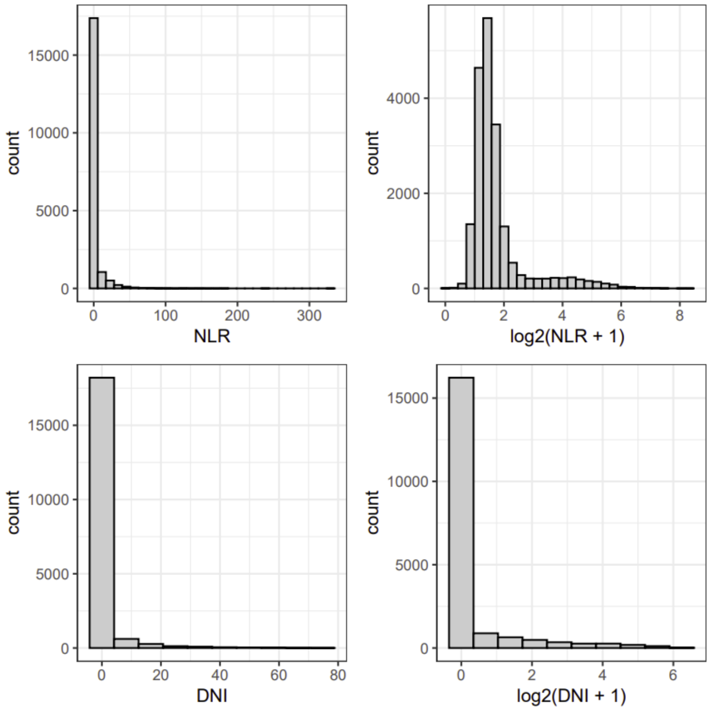

Reference

Ahn, C., Kim, W., Lim, T.H., Cho, Y., Choi, K.S., and Jang, B.H. (2018). The delta neutrophil index (DNI) as a prognostic marker for mortality in adults with sepsis: a systematic review and meta-analysis. Sci Rep *8*, 6621.

Assinger, A., Schrottmaier, W.C., Salzmann, M., and Rayes, J. (2019). Platelets in Sepsis: An Update on Experimental Models and Clinical Data. Frontiers in Immunology *10*.

Bateman, R.M., Sharpe, M.D., Singer, M., and Ellis, C.G. (2017). The Effect of Sepsis on the Erythrocyte. Int J Mol Sci *18*, 1932.

Bone, R.C., Balk, R.A., Cerra, F.B., Dellinger, R.P., Fein, A.M., Knaus, W.A., Schein, R.M., and Sibbald, W.J. (1992). Definitions for sepsis and organ failure and guidelines for the use of innovative therapies in sepsis. The ACCP/SCCM Consensus Conference Committee. American College of Chest Physicians/Society of Critical Care Medicine. Chest *101*, 1644-1655.

Butcher, S., Chahel, H., and Lord, J.M. (2000). Review article: ageing and the neutrophil: no appetite for killing? Immunology *100*, 411-416.

Celik, I.H., Arifoglu, I., Arslan, Z., Aksu, G., Bas, A.Y., and Demirel, N. (2019). The value of delta neutrophil index in neonatal sepsis diagnosis, follow-up and mortality prediction. Early Hum Dev *131*, 6-9.

Cha, Y.S., Lee, K.H., Lee, J.W., Choi, E.H., Kim, H.I., Kim, O.H., Cha, K.C., Kim, H., and Hwang, S.O. (2016). The use of delta neutrophil index and myeloperoxidase index as diagnostic predictors of strangulated mechanical bowel obstruction in the emergency department. Medicine *95*.

Cha, Y.S., Yoon, J.M., Jung, W.J., Kim, Y.W., Kim, T.H., Kim, O.H., Cha, K.C., Kim, H., Hwang, S.O., and Lee, K.H. (2015). Evaluation of usefulness of myeloperoxidase index (MPXI) for differential diagnosis of systemic inflammatory response syndrome (SIRS) in the emergency department. Emerg Med J *32*, 304-307.

de Jager, C.P., van Wijk, P.T., Mathoera, R.B., de Jongh-Leuvenink, J., van der Poll, T., and Wever, P.C. (2010). Lymphocytopenia and neutrophil-lymphocyte count ratio predict bacteremia better than conventional infection markers in an emergency care unit. Crit Care *14*, R192.

Farkas, J.D. (2020). The complete blood count to diagnose septic shock. J Thorac Dis *12*, S16-S21.

Guclu, E., Durmaz, Y., and Karabay, O. (2013). Effect of severe sepsis on platelet count and their indices. Afr Health Sci *13*, 333-338.

Huang, Z., Fu, Z., Huang, W., and Huang, K. (2020). Prognostic value of neutrophil-to-lymphocyte ratio in sepsis: A meta-analysis. Am J Emerg Med *38*, 641-647.

Jiang, Y., Jiang, F.Q., Kong, F., An, M.M., Jin, B.B., Cao, D., and Gong, P. (2019). Inflammatory anemia-associated parameters are related to 28-day mortality in patients with sepsis admitted to the ICU: a preliminary observational study. Ann Intensive Care *9*, 67.

Kim, H., Kong, T., Chung, S.P., Hong, J.H., Lee, J.W., Joo, Y., Ko, D.R., You, J.S., and Park, I. (2017). Usefulness of the Delta Neutrophil Index as a Promising Prognostic Marker of Acute Cholangitis in Emergency Departments. Shock *47*, 303-312.

Liu, X., Shen, Y., Wang, H., Ge, Q., Fei, A., and Pan, S. (2016). Prognostic Significance of Neutrophil-to-Lymphocyte Ratio in Patients with Sepsis: A Prospective Observational Study. Mediators Inflamm *2016*, 8191254.

Martin-Loeches, I., Guia, M.C., Vallecoccia, M.S., Suarez, D., Ibarz, M., Irazabal, M., Ferrer, R., and Artigas, A. (2019). Risk factors for mortality in elderly and very elderly critically ill patients with sepsis: a prospective, observational, multicenter cohort study. Ann Intensive Care *9*, 26-26.

Park, B.H., Kang, Y.A., Park, M.S., Jung, W.J., Lee, S.H., Lee, S.K., Kim, S.Y., Kim, S.K., Chang, J., Jung, J.Y*., et al.* (2011). Delta neutrophil index as an early marker of disease severity in critically ill patients with sepsis. BMC Infect Dis *11*, 299.

Park, S.Y., Lee, J.S., Oh, J., and Park, J.Y. (2020). Delta neutrophil index as a predictive and prognostic factor for Candidemia patients: a matched case-control study. BMC Infect Dis *20*, 396.

Piagnerelli, M., BOUDJELTIA, K.Z., Gulbis, B., Vanhaeverbeek, M., and VINCENT, J.L. (2007). Anemia in sepsis: the importance of red blood cell membrane changes. Transfusion Alternatives in Transfusion Medicine *9*, 143-149.

Seok, Y., Choi, J.R., Kim, J., Kim, Y.K., Lee, J., Song, J., Kim, S.J., and Lee, K.A. (2012). Delta neutrophil index: a promising diagnostic and prognostic marker for sepsis. Shock *37*, 242-246.

Vardon-Bounes, F., Ruiz, S., Gratacap, M.-P., Garcia, C., Payrastre, B., and Minville, V. (2019). Platelets Are Critical Key Players in Sepsis. Int J Mol Sci *20*, 3494.

Weiskopf, D., Weinberger, B., and Grubeck-Loebenstein, B. (2009). The aging of the immune system. Transpl Int *22*, 1041-1050.

Wenisch, C., Patruta, S., Daxböck, F., Krause, R., and Hörl, W. (2000). Effect of age on human neutrophil function. J Leukoc Biol *67*, 40-45.

Yonezawa, K., Horie, O., Yoshioka, A., Matsuki, S., Tenjin, T., Tsukamura, Y., Yoneda, M., Shibata, K., Koike, Y., Nomura, T*., et al.* (2010). Association between the neutrophil myeloperoxidase index and subsets of bacterial infections. Int J Lab Hematol *32*, 598-605.
